# Fear of Progression in Pediatric Cancer Patients and Their Parents: Trajectories, Prevalence, and Correlates Across Acute Treatment and Follow‐Up Care

**DOI:** 10.1002/pon.70514

**Published:** 2026-06-06

**Authors:** Jessy Herrmann, Kristina Herzog, Julia Martini, Anja Santel, Leonard Konstantin Kulisch, Jörn‐Sven Kühl, Florian Schepper

**Affiliations:** ^1^ Department of Pediatric Oncology Haematology and Haemostaseology Leipzig University Leipzig Germany; ^2^ Registered Association for parents of children with cancer Leipzig Leipzig Germany; ^3^ Department of Paediatric Oncology and Haematology University Hospital Carl Gustav Carus Dresden Dresden Germany; ^4^ Department of Psychiatry and Psychotherapy Faculty of Medicine of the Technische Universität Dresden Dresden Germany; ^5^ Mental Health Research and Treatment Centre (FBZ), Faculty of Psychology Ruhr‐University Bochum and German Center for Mental Health (DZPG) Bochum Germany; ^6^ Department of Pediatric Psychiatry, Psychotherapy, and Psychosomatics Leipzig University Leipzig Germany

**Keywords:** family‐centered care, fear of progression, longitudinal study, parental distress, pediatric oncology, psychosocial burden

## Abstract

**Purpose:**

Fear of progression (FoP) represents a significant psychological burden for pediatric cancer patients and their parents. This study investigates FoP levels across acute treatment (AcT) and follow‐up care (FuC) and examines trajectories over time, associated sociodemographic factors, and parent‐child associations. It also proposes clinically relevant thresholds for psychosocial care.

**Methods:**

A total of 171 patient–parent dyads participated in a cross‐sectional and longitudinal study. Children aged 7–18 and one parent per child completed the Fear of Progression Questionnaire—Short Form (FoP‐Q‐SF). Data were collected during AcT (two time points) and FuC. Statistical analyses included nonparametric tests and correlation analyses.

**Results:**

FoP levels were significantly lower during FuC compared to AcT for both children and parents, and parental FoP decreased over the 1‐year follow‐up period. Parents consistently showed higher FoP than their children, and a significant parent–child correlation emerged in FuC. FoP was higher in girls and was positively associated with child age and negatively associated with parent age. Using suggested thresholds, 57.8% of parents showed dysfunctional FoP. Among children, 45.2% reported low, 40% moderate, and 14.8% high FoP.

**Conclusion:**

Although levels of FoP are lower in FuC than in AcT, they remain a prevalent burden—particularly for younger parents, older children, and girls. The observed parent‐child associations highlight the need for family‐oriented psychosocial care. A proposed three‐stage cut‐off (low, moderate, high FoP) may guide clinical decision‐making and support tailored treatment strategies. Routine screening and preventive approaches are recommended to mitigate FoP and its potential intergenerational transmission.

**Trial Registration:**

The study has been pre‐registered at the German Clinical Trials Register (DRKS 00022034, registered 29^th^ of June 2020) and at the Open Science Foundation (https://osf.io/fuahc).

## 1 Background

Fear of disease progression, recurrence, or metastasis is presumed to represent a substantial psychological burden for pediatric cancer patients and their families. However, empirical evidence to date has largely been derived from adult oncology populations [1, 2, 3], and systematic investigation within pediatric oncology remains scarce [4]. While the constructs Fear of Cancer Recurrence (FCR) and Fear of Progression (FoP) have often been used interchangeably in the literature [5], recent empirical findings suggest that they represent related but distinct phenomena. According to Coutts‐Bain et al. [6], FCR is more closely associated with concerns about the cancer returning after treatment, whereas FoP encompasses broader anxieties about the illness worsening or spreading. These distinctions are also reflected in the psychometric properties and content of commonly used assessment tools [6]. In the present study, we focus on research employing *the Fear of Progression Questionnaire—Short Form* (FoP‐Q‐SF) [7, 8], which is specifically designed to capture FoP in oncological contexts.

FoP is not entirely dysfunctional, a certain level of FoP may even promote adherence to medical recommendations and compliance with treatment protocols [5]. Nevertheless, elevated levels of FoP in both patients and their relatives are associated with increased psychological distress, anxiety, depression and posttraumatic stress symptoms [5, 9, 10, 11], as well as with reduced health‐related quality of life (HRQoL; [1, 12, 13]).

### 1.1 Prevalences of FoP

Clever et al. [10] investigated a sample of *N* = 181 parents whose children were either undergoing acute treatment or were in (long‐term) follow‐up care. Their findings indicated that 61.8% of parents show dysfunctional FoP. In a subsequent study involving *N* = 516 parents surveyed after the competition of their child's acute treatment, 48% were found to have dysfunctional levels of FoP [12]. A Chinese validation study using the same instrument reported even higher prevalence, with 75.1% of *N* = 285 parents of children in active treatment exceeding the clinical cut‐off [13]. Across these studies, a cut‐off score ≥ 34 on the FoP‐Q‐SF/P was used to indicate dysfunctional FoP.

Luz et al. [11] adapted the *Fear of Progression Questionnaire Short‐Form* for Children (FoP‐Q‐SF/C) and applied it to a sample of *N* = 32 children aged 10–17 years undergoing acute treatment or (long‐term) follow‐up care. An empirical three‐stage cut‐off (M ± 1 SD) was used, classifying 21.8% of the children as highly burdened, 62,5% as moderately burdened and 15.5% as experiencing low burden. Zhang et al. [13] examined a sample of *N* = 102 children in acute treatment using a Chinese version of the questionnaire and applied a two‐stage cut‐off derived from the 5‐point Likert response scale. They reported that 43.74% of the children were highly distressed; however, this result should be interpreted with caution, due to unclear methodological details.^1^ A validation of the German FoP‐Q‐SF/C was presented by Schepper et al. [14].

To date, there is no established consensus regarding a clinically meaningful threshold for FoP in pediatric oncology. The cut‐off score proposed for identifying dysfunctional FoP in parents [10] was originally derived from adult oncology patients' self‐reported need for psychological support [15]. This threshold was subsequently applied to relatives of cancer patients, despite evidence that these groups tend to report substantially higher levels of FoP. For instance, the prevalence of dysfunctional FoP has been reported at 16.7% in adult oncology patients [8], compared to 47%–51% in their spouses [3] and 48–75,1% in parents of children with cancer [10, 12, 13]. These differences suggest that the need for psychosocial intervention may vary considerably across groups and that a uniform threshold may not be appropriate. Clever et al. [16] explored expert perspectives by surveying 77 professionals in pediatric oncology, who estimated that approximately 40% of parents exhibit a need for psychosocial intervention. Research on the prevalence and clinical relevance of dysfunctional FoP in pediatric patients themselves remains in its early stages. Therefore, in addition to the proposed cut‐offs, we employed pragmatic thresholds based on scale levels for both parents and children.

### 1.2 Variables Associated With FoP

FoP is characterized as a persistent fear [17], and it has been observed in both pediatric cancer patients and their parents in acute treatment [9, 10, 11, 13, 18] as well as (long‐term) follow‐up care [9, 10, 11, 12]. While some evidence suggests that FoP tend to be more pronounced during acute treatment compared to follow‐up phases [9], findings regarding its associations with time since diagnosis remain inconsistent [9, 10, 13].

Several medical and psychosocial factors have been linked to elevated levels of FoP. For parents these include having a child with central nervous system tumor (CNS tumor) in comparison to Leukemia, the presence of depressive symptoms [12], lower levels of self‐compassion, poor sleep quality, severe posttraumatic stress symptoms [13] and heightened awareness of the child's physical symptoms [10]. In children, both the presence of a tumor of the reproductive system and the parents' perception of whether their child needed psychological care were associated with increased FoP [18].

Sociodemographic variables may also play a role in the manifestation of FoP. Luz et al. [11] found no significant gender differences in FoP among pediatric patients. However, findings concerning parental gender differences remain inconsistent: while some studies report higher FoP in mothers compared to fathers [19], others did not observe significant differences [9, 13, 20]. Furthermore, Schepper et al. [9] identified a significant negative correlation between parental age and FoP, whereas no significant correlation was found with the child's age at diagnosis.

### 1.3 Study Aims

This study investigates levels of FoP in pediatric cancer patients and their parents during acute treatment (AcT) and follow‐up care (FuC), with the aim of assessing psychosocial burden across different treatment phases. As previous results on associated sociodemographic variables were inconsistent, we analyzed associations with children's and parental age and gender to better identify patients at risk. Psychosocial factors, although relevant, were not included as their consideration would have exceeded the scope of this study. By analyzing correlations between children's and parents' FoP, we aim to contribute to a better understanding of FoP as a family‐level phenomenon and to inform the development of targeted interventions and preventive strategies. In addition, we address the question of clinically relevant thresholds for FoP, above which psychological intervention may be indicated.

Based on data from pediatric cancer patients and their parents in AcT and FuC, the following research questions will be examined:

(RQ1) What are the levels of FoP in pediatric cancer patients and their parents, and do they differ by (a) treatment status, (b) age and (c) gender?

(RQ2) What are the prevalences of (a) dysfunctional FoP in parents based on the suggested threshold (≥ 34) and (b) low, moderate and high FoP in children based on scale‐level thresholds?

(RQ3) Does the level of FoP in children and parents change over the course of 1 year?

(RQ4) Do FoP levels in children and parents (a) differ and (b) correlate within patient—parent dyads?

## 2 Methods

### 2.1 Design and Procedure

The present study is part of a larger research project between 2019 and 2022 with two parts: Study 1 is a prospective‐longitudinal study with two data points (T1 and T2) one year apart in AcT, while study 2 is a cross‐sectional study with one data point in FuC. Ethics approval was obtained from the ethics committee of the Technische Universität Dresden (EK‐514112015) and the Universität Leipzig (366/14‐ff). The project has been pre‐registered at the German Clinical Trials Register (DRKS‐00022034) and at the Open Science Foundation (https://osf.io/fuahc).

Children in study 1 were recruited in the acute wards for pediatric oncology at the university hospitals in Dresden and Leipzig, Germany. Children in study 2 were recruited in the parents' associations in Dresden and Leipzig (Sonnenstrahl e.V. Dresden, Elternhilfe e.V. Leipzig). One parent per child was also invited to participate. After obtaining written informed consent of parents and children they completed measures of FoP. Although the overall study included children aged 4–18 years, analyses of FoP self‐reports were limited to children aged ≥ 7 years due to the availability and developmental suitability of validated instruments. Parent‐reported FoP was collected for parents of all participating children, including those aged 4–6 years. In addition, information on sociodemographic and medical characteristics of child and parent was obtained from the parent. In study 1, children and parents were contacted by phone and asked to participate for a second time 1 year later (T2). Questionnaires for older children from the age of 12 years and their parents were sent by mail, while younger children completed the questionnaire at the hospital after a scheduled control examination. A researcher (KH) was present to assist with any questions. Inclusion criteria for pediatric patients were (1) for studies 1 and 2: pediatric oncology patients aged 4–18 years, (2) for study 1: first diagnosis at least 1 month ago, (3) for study 2: first diagnosis at least 2 years ago; being off active treatment. Inclusion criteria for parents were (1) being caregiver of a participating pediatric patient and (2) ability to provide informed consent. Exclusion criteria for both children and parents were (1) insufficient ability to understand and complete the questionnaire or participate in the interview due to language barriers or cognitive/mental impairment (as assessed by psychosocial staff), and (2) palliative care. In addition to the predefined inclusion and exclusion criteria, case managers were consulted for each family to evaluate whether, considering their individual circumstances (e.g., episodes of acute exceptional burden) it was appropriate to approach them regarding study participation. When necessary, recruitment was postponed to a later date.

### 2.2 Sample

**FIGURE 1:**
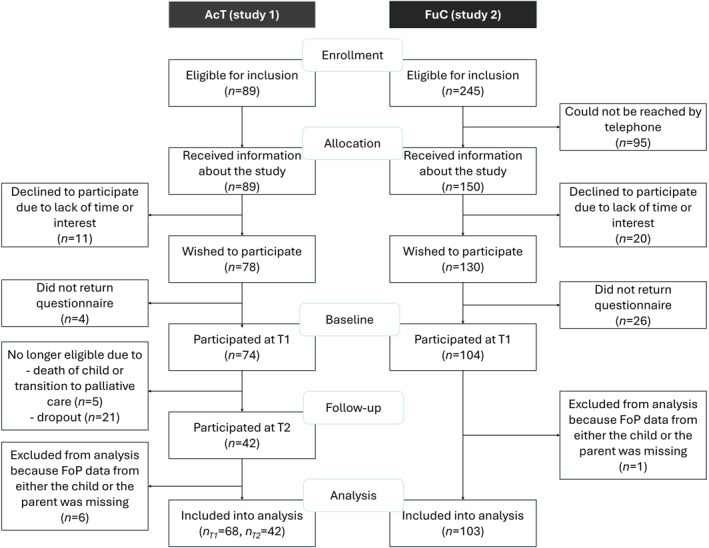
CONSORT diagram (adapted, non‐randomized) on participation in study 1 (dyads with a child in AcT and FuC) and 2 (dyads with a child in FuC).

The flow chart on participation is shown in Figure 1. In total, *n* = 334 patient–parent dyads were eligible for inclusion and were informed about the study by our staff, and *n* = 178 dyads agreed to participate. Of these, *n* = 171 dyads were included in the analysis, as FoP scores were available for at least one member of the dyad (either the child or the parent). In *n* = 110 cases FoP scores were available from both parent and child. In *n* = 56 cases, FoP scores were available only from the parent (mainly due to the child being too young to complete the FoP‐Q‐SF) and in *n* = 5 cases, FoP scores were available only from the child. A total of *n* = 68 dyads in Study 1 and *n* = 103 dyads in Study 2 were included in the analyses. In Study 1, *n* = 42 dyads also participated at the one‐year follow‐up (T2). Of these, *n* = 5 children were still in acute treatment, whereas the remaining *n* = 37 children had transitioned to follow‐up care. Overall, *n* = 21 dyads discontinued participation (reasons: lack of time, inability to reach families via phone or email), and *n* = 5 children either entered palliative care or died during the study period. Drop‐out analyses revealed no significant differences between completers and non‐completers, except for age; children who completed the study were younger than those who dropped out (see Supporting Information S1: Appendix 1). Sociodemographic and illness characteristics are summarized in Table 1.

**TABLE 1:** Sample characteristics.

		Study 1 (*n* = 68)	Study 2 (*n* = 103)
Children			
Gender (*n*, %)			
	Male	29 (42.6)	66 (64.1)
	Female	39 (57.4)	37 (35.9)
	Diverse		
Age in years (*M,* SD)		10.19 (4.0)	12.37 (3.6)
Diagnosis (*n*, %)			
	Leukemia	25 (36.8)	41 (39.8)
	Lymphoma	12 (17.6)	11 (10.7)
	Tumor of the central nervous system	11 (16.2)	27 (26.2)
	Other solid tumors^a^	20 (29.4)	20 (19.4)
	Langerhans cell histiocytosis (LCH)	0 (0.0)	4 (3.9)
Time since diagnosis in months (*M,* SD)		7.5 (15.3)	78.0 (43.9)
Treatment modality (*n*, %—*multiple responses possible)*			
	Chemotherapy	62 (91.2)	90 (87.4)
	Radiotherapy	8 (11.8)	24 (23.3)
	Surgical measures	27 (39.7)	41 (39.8)
	Hematopoietic stem cell transplant	2 (2.9)	12 (11.7)
	Other^b^	0 (0.0)	4 (3.9)
Combination of treatment modalities			
	Unimodal	42 (61.8)	58 (56.3)
	Multimodal	26 (38.2)	45 (43.7)
Parent			
Participating parent (*n*, %)			
	Mother	59 (86.8)	83 (80.6)
	Father	8 (11.8)	17 (16.5)
	No parent participated	1 (1.4)	3 (2.9)
Age in years (*M,* SD)		38.8 (6.1)	43.1 (6.2)

^a^for example bone tumor, dysgerminoma.

^b^ for example immune therapy, proton beam irradiation.

### 2.3 Measures

#### 2.3.1 Fear of Progression

All parents completed the German adaptation of the Fear of Progression Questionnaire—Short Form for parents (FoP‐Q‐SF/P; [10]). Children from the age of 7 years completed the adaption for children (FoP‐Q‐SF/C; [11, 14]). The FoP‐Q measures FoP using 12 items with a five‐point Likert‐scale ranging from 1 (never) to 5 (very often). Children and parents were asked about their own FoP, respectively (e.g., “I get anxious when I think that my child's disease may progress.” vs. “I get anxious when I think that my illness may progress.”). The continuous FoP score is calculated by summing the items, with higher scores indicating higher FoP. The FoP‐Q‐SF/P and FoP‐Q‐SF/C showed good internal consistency, convergent and discriminant validity [10, 11]. In the present study, internal consistency for the FoP‐Q‐SF/P (*α* = 0.90) and FoP‐Q‐SF/C (*α* = 0.87) was good.

For children's FoP a threshold based on scale level of the FoP‐Q‐SF was used: Thus the answers (1) “never”, and (2) “rare” (sum between 12 and 24) are considered low, (3) “sometimes” (sum between 25 and 36) is considered moderate and (4) “often” and (5) “very often” (sum between 37 and 60) are considered as high levels of FoP [11, 18]. For parents we used the suggested cut‐off ≥ 34 for dysfunctional FoP [10, 15] and also applied the described the threshold based on scale level.

### 2.4 Statistical Analysis

For the present analyses, data from study 1 and study 2 were used with a defined analytical separation. Cross‐sectional analyses (RQ1 and RQ2) were conducted using a combined total sample consisting of baseline data (T1) from study 1 and data from study 2. No longitudinal inferences were drawn from the combined cross‐sectional sample. Longitudinal analyses (RQ3) were restricted exclusively to participants from study 1 who provided data at both T1 and T2. Dyadic analyses (RQ4) included all available parent–child pairs with concurrent FoP assessments at the same measurement occasion. Analyses were conducted separately for each available time point and study and were not interpreted longitudinally.

Analyses were performed using IBM SPSS Statistics 27.0 [21].

Descriptive statistics of FoP scores (RQ1 and RQ2) were calculated. Nonparametric tests (Kendall‐Tau correlations, Mann‐Whitney‐*U*‐tests, and Kruskall‐Wallis tests) were used to analyze differences in FoP by (a) treatment status, (b) age and (c) gender as children's FoP scores were not normally distributed.

Wilcoxon signed‐rank tests were used to analyze longitudinal course of FoP in study 1 (RQ3) as well as to analyze differences between children's and parental FoP within parent‐child dyads (RQ4a). Kendall‐Tau correlations were calculated to analyze correlations between children's and parental FoP scores within dyads (RQ4b).

For all tests *p* < 0.05 was considered statistically significant.

Sensitivity analyses were conducted using G*Power 3.1.9.7. to determine the *minimum detectable effect sizes,* assuming *α* = 0.05 (two‐tailed) and power (1−*β*) = 0.80. These analyses were performed for all inferential statistical procedures except the descriptive prevalence estimates in Research Question 2. Results indicated that the available sample sizes allowed detection of small‐to‐medium effects in larger cross‐sectional analyses, while only medium‐sized effects could be detected in the smaller longitudinal and dyadic samples. Detailed results are provided in Supporting Information S1: Appendix 2.

## 3 Results

### 3.1 Descriptive Statistics of FoP for Children and Parents, Differences by Treatment Status, Age and Gender (RQ1)

**FIGURE 2:**
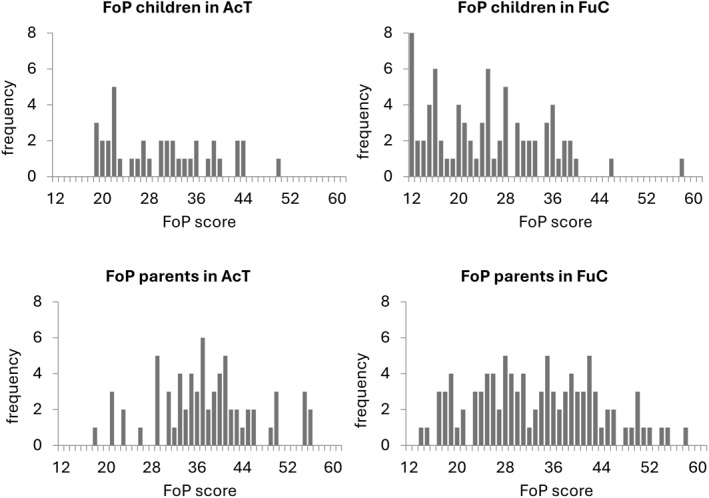
Distribution of parent and child FoP in AcT (study 1/T1) and FuC (study 2).

Unless otherwise stated, results for Research Questions 1 and 2 refer to analyses of the combined cross‐sectional sample (study 1/T1 and study 2). Descriptives for children and parents in study 1 (T1: AcT; T2: AcT and FuC) and study 2 (FuC) as well as the overall sample (study 1/T1 and study 2) are summarized in Table 2 and the results of other studies using the same instrument (FoP‐Q‐SF/P or C) are shown alongside for comparison purposes. Figure 2 shows distributions of children's and parental FoP scores in AcT (study 1/T1) and FuC (study 2).

**TABLE 2:** Descriptive statistics of FoP in children and their parents in study 1 and 2 as well as results of other studies using the same instrument (FoP‐Q‐SF/P or C).

	Children		Parents	
	*n*	*M* (SD)	*n*	*M* (SD)
Study 1				
T1 (AcT, TsD in years *M* = 0.6, SD = 1.3)	38	30.1 (8.6)	67	37.7 (8.8)
T2 (AcT and FuC, TsD in years *M* = 1.8, SD = 1.6)	27	27.7 (8.6)	41	34.3 (8.7)
Study 2				
T1 (FuC, TsD in years *M* = 6.5, SD = 3.7)	77	24.6 (9.6)	99	33.3 (10.3)
Overall sample (study 1/T1, study 2)	115	26.4 (9.6)	166	35.0 (9.9)
Other studies using the FoP‐Q‐SF				
Luz et al. 2000 (AcT and FuC) (TsD in years: *M* = 3.1, SD = 3)	32	28.2 (9.6)		
Zhang et al. 2023 (AcT) (no TsD reported)	102	32.7^a^		
Clever et al. 2018 (AcT and FuC)(TsD in years *M* = 2.5, SD = 2.2)			181	36.0 (9.8)
Peikert et al. 2021 (FuC)(TsD in years *M* = 1.8, SD = 1.8)			516	33.8 (9.4)
Yang et al. 2022 (AcT)(TsD in years *M* = 0.5, SD = 0.6)			285	40.0 (9.2)

Abbreviations: AcT, Acute treatment; FuC, Follow‐up care; TsD, Time since diagnosis.

^a^ no SD is reported.

FoP levels in children were not normally distributed (Shapiro‐Wilk test: *p* = 0.006) and there were several peaks in the distribution with a tendency toward low to moderate symptom severity. FoP scores of children in study 2 were significantly lower (*U* = 964.00, *p* = 0.003, *r* = 0.260; AcT: *M* = 30.1, SD = 8.6; FuC: *M* = 24.6, SD = 9.6) compared to study 1/T1. No significant association between children's FoP levels and age was observed in the overall sample (*r* = 0.079, *p* = 0.242) or in study 1/T1 (*r*_*τ*_ = −0.03, *p* = 0.780). In contrast, in study 2, a positive association emerged (*r*_*τ*_ = 0.19, *p* = 0.022), indicating that older children reported higher FoP. However, the sensitivity analyses showed that none of these analyses were powered to detect effects of this magnitude. Regarding gender, significant differences in children's FoP levels were observed in the overall sample (*U* = 2.607, *p* = 0.009, *r* = 0.243), with girls reporting higher FoP than boys (girls: *M* = 28.7, SD = 8.8; boys: *M* = 24.6, SD = 9.9), and likewise in study 2 (*U* = −2.54, *p* = 0.011, *r* = 0.289; girls: *M* = 27.7, SD = 8.8; boys: *M* = 22.7, SD = 9.6). In study 1/T1, no gender differences were found (*U* = −0.17, *p* = 0.883; girls: *M* = 29.9, SD = 8.9; boys: *M* = 30.5, SD = 8.5), however, the sensitivity analyses indicated that only medium‐to‐large effects could be detected in this subsample.

Parental FoP levels were normally distributed (Shapiro‐Wilk test: *p* = 0.115). In study 1/T1, there was a clear peak in frequency (at 35–40) indicating a tendency to moderate distress. Parental FoP scores in study 2 were significant lower (*U* = 2478.00, *p* = 0.006, *r* = 0.214; study 1/T1: *M* = 37.7, SD = 8.8; study 2: *M* = 33.3, SD = 10.3) than in study 1/T1. A significant association between age and parental FoP levels was observed in the overall sample (*r* = −0.157, *p* = 0.004), indicating that younger parents reported higher FoP. No significant age–FoP associations emerged in study 1/T1 (*r*_*τ*_ = −0.16, *p* = 0.072) or study 2 (*r*_*τ*_ = −0.10, *p* = 0.160), although these analyses were underpowered to detect effects of this magnitude. Regarding gender, no differences in FoP were observed in the overall sample (*U* = −0.054, *p* = 0.957; mothers: *M* = 35.1, SD = 10.0; fathers: *M* = 34.8, SD = 9.3) nor within study 1/T1 (*U* = −0.01, *p* = 0.992) or study 2 (*U* = 0.30, *p* = 0.766). However, the sensitivity analyses showed that only medium to large effects were detectable, indicating that the analyses were insufficiently powered to identify small gender differences. Additionally, these findings should be interpreted cautiously due to the small number of participating fathers.

### 3.2 Prevalences of FoP in Children and Parents (RQ 2)

**FIGURE 3:**
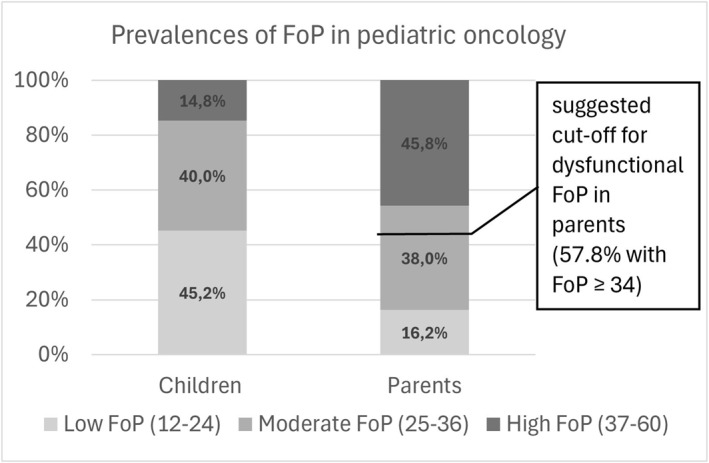
Prevalences of low, moderate and high FoP in children (*n* = 115) and parents (*n* = 166) using the threshold based on scale level, and the cut‐off for dysfunctional FoP in parents (≥ 34) suggested by Clever et al. [14].

Using a threshold based on scale level as described in the methods section, 34.2% of the children in study 1/T1 (*n* = 38) showed low FoP, 42.1% showed moderate FoP and 23.7% showed high FoP. In study 2 (*n* = 77), 50.5% of the children showed low FoP, 39.0% showed moderate FoP and 10.4% showed high FoP. Figure 3 presents the proportions for the entire sample (*n* = 115).

Using the cut‐off ≥ 34 suggested by Clever et al. [10], 70.1% of parents in study 1/T1 (*n* = 67), 49.5% in study 2 (*n* = 99) and 57.8% in the whole sample (*n* = 166) showed dysfunctional FoP. For comparative purposes we applied the above‐mentioned threshold based on scale level for the sample of parents: 9.0% of the parents in study 1/T1 showed low FoP, 34.3% showed moderate FoP and 56.7% showed high FoP. In study 2, 21.2% of the parents showed low FoP, 40.4% showed moderate FoP and 38.4% showed high FoP. Figure 3 presents the proportions for the entire sample (*n* = 166).

### 3.3 Trajectories of FoP (RQ 3)

Longitudinal results for RQ 3 are based exclusively on participants from study 1 with data at both T1 and T2. Wilcoxon signed‐rank tests showed that parental FoP scores in study 1 (T1 to T2) decreased significantly over 1 year (*Z* = −2.21, *p* = 0.027), while children's FoP scores did not change significantly (*Z* = −0.50, *p* = 0.615). Given the minimal detectable effect size (dz = 0.56), smaller changes in children's FoP may have been below the study's sensitivity threshold and therefore remained statistically undetectable.

### 3.4 Associations Between Children's and Parental FoP Scores (RQ 4)

Results for Research Question 4 are derived from dyadic analyses of parent–child pairs with concurrent FoP assessments at the same measurement occasion. Analyses were conducted separately for each time point and study.

Wilcoxon signed‐rank tests revealed differences in FoP scores between children and parents in study 1 with medium to large effect size (T1: *n* = 37, *Z* = −3.35, *p* = 0.001, *r* = 0.55; T2: *n* = 26, *Z* = −2.96, *p* = 0.003, *r* = 0.58) and study 2 (*n* = 73, *Z* = −5.64, *p* < 0.001, *r* = 0.66; [22]), with parents reporting higher FoP compared to their children.

Kendall‐Tau correlations indicated no significant association in FoP scores between children and parents in study 1, neither at T1 (*r*_*τ*_ = 0.11, *p* = 0.351) nor 1 year later at T2 (*r*_*τ*_ = 0.18, *p* = 0.215). However, given the detectable effect size thresholds (dz = 0.44 at T1 and dz = 0.52 at T2), only small‐to‐medium (T1) to medium‐to‐large (T2) parent–child associations would have been identifiable; thus, weaker correlations may have gone undetected. In study 2, children's and parental FoP scores were positively correlated (*r*_*τ*_ = 0.21, *p* = 0.010).

## 4 Discussion

To our knowledge, this is the first study to investigate fear of progression (FoP) in pediatric patients and their parents across both acute treatment and follow‐up care, while also integrating longitudinal data and dyadic analyses within a sample of 171 families.

### 4.1 Differences Across Treatment Settings

One of our key findings concerns the differences in FoP across treatment settings, considered alongside the longitudinal data. First, participants in study 2 (FuC) reported significantly lower levels of FoP than those in study 1/T1 (AcT), both among patients and their parents (RQ1a). This cross‐sectional finding should be interpreted considering the limitations of the study sample, which may restrict external validity. Second, in line with this pattern, longitudinal data from study 1 showed a significant decrease in parental FoP over 1 year, whereas children's FoP did not change significantly (RQ2). This result should be interpreted cautiously, as sensitivity analyses indicated that the longitudinal analysis of children's FoP was underpowered to detect small effects (minimal detectable effect size: dz = 0.56), and selective dropout of older children may have further reduced sensitivity to change. Therefore, small changes in children's FoP cannot be ruled out. At the same time, no established minimal clinically important difference (MCID) is currently available for the FoP‐Q‐C/SF, limiting the ability to formally evaluate the clinical relevance of small observed changes. Thus, the apparent stability in children's FoP may reflect either true temporal stability or changes of limited magnitude that could not be reliably detected and whose clinical relevance cannot yet be determined.

While studies in adult oncology have demonstrated the temporal stability of the related construct FCR [1] we assume that FoP may decline with transitions in the treatment setting, as previously proposed by Schepper et al. [9] and Clever et al. [10]. This assumption is supported by findings on cancer‐related fears [23] and other psychosocial outcomes (e.g., illness perception; [24]). FoP may be particularly pronounced during AcT, a phase often marked by heightened psychosocial burden [25], uncertainty regarding prognosis, and distress related to somatic symptoms and potential long‐term consequences of the disease and its treatment. In contrast, the follow‐up phase may be characterized by positive developments (e.g., recovery from treatment side effects, reassuring follow‐up results) which could contribute to a reduction of problems [26] and may cause a decrease in FoP. Additionally, the gradual normalization of everyday family life, reintegration into social environments (e.g., school, work), and increased confidence in future planning [27] may further alleviate FoP.

### 4.2 Associations in Parent‐Child Dyads

A second key set of findings relates to the parent–child dyads (RQ4), which provide important insight into how FoP may be shared within a family. A significant correlation (RQ4b) between parents' and children's FoP scores emerged in study 2, whereas no such relationship was found in study 1. Importantly, sensitivity analyses showed that only study 2 was sufficiently powered, warranting cautious interpretation of the non‐significant findings in study 1. Age‐related attrition may be another factor to consider when interpreting parent–child associations in study 1.

Nevertheless, the overall pattern aligns with previous research showing that parents and children can mutually influence each other's fears and illness‐related cognitions [28, 29]. For example, when parents exhibit elevated FoP and adopt controlling coping strategies—such as frequent monitoring of their child's physical symptoms—children may internalize these fears through observation and emotional attunement. The absence of a correlation in Study 1 can be understood in light of evidence indicating that during the acute phase of pediatric cancer treatment, parents tend to prioritize supporting and protecting their child while regulating, containing, or postponing their own fears [30]. This role‐related emotional regulation is suggested to be associated with increased caregiving demands, decision‐making responsibility, and child‐focused self‐efficacy, and may reduce both the observability and communicative sharing of fears within the parent–child dyad [31, 32]. Concepts on protective emotional regulation—such as the concept of *double protection*, in which parents and children mutually attempt to shield one another from emotional distress—may offer a useful conceptual lens for understanding the reduced observable association in self‐reported fears [33]. As treatment intensity decreases and families transition into follow‐up care (FuC), such constraints may ease, allowing experiences from the acute phase to be increasingly processed at the family level and integrated into a shared illness narrative, which may foster greater emotional alignment and communication, consistent with the correlation observed in study 2 [34].

Additionally, parents consistently reported higher FoP levels than their children (RQ4a). This aligns with previous research [9, 10, 19] and likely reflects the heightened sense of responsibility and ongoing vigilance that parents maintain throughout their child's illness trajectory, which may increase their distress and reduce opportunities for self‐care.

Together, these findings underscore the importance of early psychosocial interventions that support parents in developing adaptive coping strategies. Such interventions may not only alleviate parental distress but also help prevent the intergenerational transmission of fear‐related responses to illness.

### 4.3 Overall FoP Levels and Associations With Sociodemographic Variables

Parental FoP levels in our sample were comparable to those reported in previous studies [10, 12, 13]. However, children showed slightly lower FoP levels than those documented in earlier research [11, 18]. Our findings regarding associations between FoP and sociodemographic variables do not offer a conclusive explanation for inconsistencies observed in previous research. The higher FoP levels found in older children and girls (RQ1b/c) is in line with theoretical frameworks and may be attributed to their advanced cognitive and emotional development, which enables a deeper understanding of the illness and its potential progression, as well as a greater capacity to reflect on its implications for their future lives [28]. This is particularly relevant for girls, who may already begin to consider future motherhood, as highlighted by Cunningham et al. [35]. Moreover, older children and girls may possess more developed communication skills, allowing to articulate their fears [36]. Given that FoP is considered an internalizing psychological issue, the observed gender differences are consistent with broader findings indicating a higher prevalence of internalizing symptoms among females [37].

With respect to sociodemographic factors, the overall sample showed a small but significant association between younger parental age and higher FoP levels, which is in line with previous research [9]. No gender differences in FoP were observed either in the overall sample or in the individual studies. However, this finding should be interpreted with caution given the overrepresentation of mothers and the limited statistical power to detect potential differences involving fathers.

Overall, the findings suggest that parental FoP is particularly elevated during the acute treatment phase, whereas sociodemographic factors such as age and gender appear to play only a minor role and should be interpreted with caution due to limited statistical power.

### 4.4 Contribution to Clinically Relevant Thresholds

With our study, we aim to contribute to the discussion on clinically relevant thresholds for the treatment of FoP. In the absence of a validated external criterion, the commonly used cut‐off for dysfunctional FoP in parents [10] was originally derived from the intervention needs of adult oncology patients [15], despite notable differences in FoP levels between patients and their relatives [3, 10]. As an interim solution, we propose pragmatic thresholds based on scale levels for both parents and children (see Figure 3), until further research—such as studies on perceived need for intervention—can establish empirically validated cut‐offs. Our findings indicate that the proportion of parents with high FoP closely aligns with the threshold for dysfunctional FoP proposed by Clever et al. [10] and is broadly consistent with expert recommendations for intervention [16]. However, the use of a single cut‐off to distinguish exclusively between functional and dysfunctional levels of FoP may insufficiently capture the spectrum of clinical need. Based on our clinical experience, we emphasize that psychological support is often warranted even in moderately burdened patients and parents. This aligns with recent evidence demonstrating that short‐term interventions may be effective in adult patients experiencing moderate levels of FoP [38]. Therefore, we advocate a three‐stage cut‐off (low, moderate, high FoP) to guide clinical decision‐making. Early intervention may be particularly important, as maladaptive coping strategies (e.g., suppression, avoidance) may exacerbate or maintain anxiety throughout the disease trajectory [39] and—as our findings suggest—FoP may be transmitted from parents to children. Conversely, early and open family communication about cancer‐related fears has been shown to be highly adaptive [40] and could be supported in targeted interventions. This clinical approach is also endorsed in the German S3 guideline on psychosocial care, which recommends intensive care for both moderately and highly distressed patients and families [41].

### 4.5 Study Limitations

This study presents several strengths like adequate sample size and data from different treatment settings. Despite these positive characteristics, the study is not without limitations.

Due to ethical considerations, in both studies, patients receiving palliative care were excluded, and in study 2 (FuC), children who were in an acute care setting due to cancer recurrence at the time of data collection were not included. These sampling decisions may result in a limitation of our data, as families facing more complex or prolonged illness trajectories are likely underrepresented in both studies, particularly in the follow‐up sample. Such trajectories are commonly associated with higher psychosocial burden, including elevated FoP [42]. Consequently, FoP levels in the present sample may be underestimated, potentially limiting the external validity of the findings. Subsequent studies should seek to incorporate families affected by extended disease courses to evaluate the robustness of the observed FoP patterns, while providing adequate ethical protection and tailored psychosocial support for these particularly vulnerable groups.

Additionally, the dropout analysis for the longitudinal data in Study 1 indicated a potential age‐related attrition bias: older children—who have been shown to experience higher FoP scores—were underrepresented in the follow‐up assessment, potentially due to age‐related shifts in priorities (e.g., greater peer orientation during adolescence). As a result, the FoP scores at Study 1/T2 may be underestimated, which could partly account for the absence of significant longitudinal change in children's FoP. Future longitudinal studies should therefore implement strategies to reduce attrition among older children to allow more robust conclusions regarding longitudinal changes in children's FoP.

Furthermore, a potential gender bias among participating parents must be acknowledged, as mothers were overrepresented—likely reflecting their more frequent involvement in hospital‐based and follow‐up care. Statistical power may therefore be limited for certain subgroup analyses, particularly for fathers. Future studies should aim to ensure more balanced recruitment of mothers and fathers to allow adequately powered gender comparisons.

The comparison between study 1 and study 2 was conducted using independently recruited samples. Consequently, no conclusions can be drawn regarding the temporal development of FoP within the same individuals across treatment phases. Moreover, the interpretation of intra‐individual change is constrained using only two measurement time points. Further research should use longitudinal data to investigate the course of FoP throughout acute treatment and long‐term follow‐up care.

Finally, interpretation of longitudinal findings is limited by the absence of an established minimal clinically important difference (MCID) for the FoP‐Q‐C/SF. Without clearly defined thresholds for clinically meaningful change, it remains difficult to determine whether small, non‐significant score changes reflect true stability or changes of limited clinical relevance. Future studies should aim to establish clinically meaningful change criteria for pediatric FoP, for example by using anchor‐based approaches in longitudinal designs.

### 4.6 Clinical Implications

We recommend the routine assessment of FoP as part of standard psychosocial screening procedures. In this context, we advocate for a three‐stage cut‐off that accounts not only for high, but also for moderate and low levels of FoP. Based on this stratification, tailored interventions are needed to reduce elevated FoP and to proactively strengthen the coping skills of patients and parents.

## 5 Conclusion

Our findings reveal that FoP is significantly higher during AcT and tends to decline over time, particularly among parents. In FuC, older children and girls reported higher FoP, likely reflecting developmental and gender‐related differences. Across both phases, parents consistently reported higher FoP than their children, and a significant parent–child correlation emerged only in FuC, possibly due to more open communication and shared illness narratives.

To support clinical decision‐making, we propose a three‐stage cut‐off (low, moderate, high FoP), as existing thresholds—derived from adult populations—may not fully reflect the needs of families in pediatric oncology. Preventive interventions are especially important, as maladaptive coping strategies may maintain FoP and promote its transmission across generations. Our findings support the integration of early, family‐centered psychosocial support into routine care, in line with national guidelines.

## Author Contributions

Conception and design of the study: Martini, Schepper. data collection: Herzog. data analysis and interpretation: Herrmann, Herzog, Santel. preparation of manuscript: Herrmann, Herzog. critical review and approval of final manuscript as submitted: Herrmann, Herzog, Santel, Martini, Kulisch, Kühl, Schepper. Herrmann and Herzog contributed equally to this work.

## Funding

This study was supported by the Stiftung Deutsche Krebshilfe under grants DKH‐70112444 and DKH‐70112467.

## Ethics Statement

This study was performed in line with the principles of the Declaration of Helsinki. It was approved by the ethics committee of the Technische Universität Dresden (EK 514112015) and the Universität Leipzig (366/14‐ff).

## Consent

Informed consent was obtained from all individual participants included in the study and from the parents of children who participated in the study.

## Conflicts of Interest

All authors declare that they have no financial relationships that might be perceived as a potential conflict of interest.

## Supporting information


Supporting Information S1

## Data Availability

The data that support the findings of this study are available from the corresponding author upon reasonable request.

## Endnotes

^1^
The analysis by Zhang et al. (2023) does not clearly specify whether high FoP was defined as a score range of 24–60 (corresponding to scale levels 3–5) or 36–60 (scale levels 4–5), which limits the interpretability of the reported prevalence.
