# Supplementary material for: Fear of Progression in Pediatric Cancer Patients and Their Parents: Trajectories, Prevalence, and Correlates Across Acute Treatment and Follow‐Up Care
Source: Psychooncology. 2026 Jun 6;35(6):e70514. doi: 10.1002/pon.70514 (PMC13242078; doi:10.1002/pon.70514)
Supplement: Supplementary file 1 — Supporting Information S1 [file PON-35-e70514-s001.docx]

Appendix 1. Analysis of dropouts (N=63): Comparison of *n*=42 patients who have completed the study (“completers”) and *n*=21 patients who have dropped out after baseline (“dropouts”)

|  | Completers  (*n*=42) | Dropouts  (*n*=21) | Statistical test |
| --- | --- | --- | --- |
| Age of child at T1 in years (M, SD) | 9.05 (3.8) | 12.14 (3.5) | *t*(61)=-3.11, *p*=.003  98% KI [-5.09, -1.10] |
| Gender of child (n, %) |  |  | *Х²*(1)=0.29, *p*=.589 |
| Male | 17 (40.5%) | 10 (37.0%) |  |
| Female | 25 (59.5%) | 11 (63.0%) |  |
|  |  |  |  |
| Diagnosis of child (n, %) |  |  | *Х²*(3)=1.62, *p*=.654 |
| Leukaemia | 15 (35.7%) | 9 (42.9%) |  |
| Lymphoma | 7 (16.7%) | 3 (14.3%) |  |
| CNS tumour | 9 (21.4%) | 2 (9.5%) |  |
| Other solid tumour | 11 (26.2%) | 7 (33.3%) |  |
|  |  |  |  |
| Responding parent (n, %) |  |  | *Х²*(1)=0.00, *p*=.953 |
| Mother | 38 (90.5%) | 18 (90.0%) |  |
| Father | 4 (9.5%) | 2 (10.0%) |  |
| Missing | 0 | 1 |  |

Note. T1=baseline.

Appendix 2. Sensitivity analyses were conducted using GPower 3.1.9.7 with α = .05 (two-tailed) and power (1−β) = .80. Reported values indicate the minimal detectable effect size given the available sample sizes. Effect size conversions from *d* to *r* follow the equation $r=\frac{d}{\sqrt{d^{2}+4}}$

Sensitivity Analysis for Research Question 1: Differences in Fear of Progression (FoP) by Treatment Status, Age, and Gender; d = Cohen’s d effect size; r = bivariate correlation coefficient

| Research  Question | Sample | Statistical Comparison | N | Sensitivity result (Equivalent) |
| --- | --- | --- | --- | --- |
| RQ1a: Treatment status (Study 1/T1 vs. Study 2) | Children | Independent groups | 38 / 77 | d = 0.56 (r=.27) |
| RQ1a: Treatment status (Study 1/T1 vs. Study 2) | Parents | Independent groups | 67 / 99 | d = 0.45 (r=.22) |
| RQ1b: Age–FoP Assoziation | Children (overall) | Correlation | 115 | r = .26 |
|  | Children  (Study 1/T1) | Correlation | 38 | r = .44 |
|  | Children (Study 2) | Correlation | 77 | r = .31 |
| RQ1b: Age–FoP Assoziation | Parents (overall) | Correlation | 166 | r = .22 |
|  | Parents (Study 1/T1) | Correlation | 67 | r = .33 |
|  | Parents (Study 2) | Correlation | 99 | r = .28 |
| RQ1c: Gender–FoP Assoziation | Children (overall) | Independent groups | 51/64 | d = .47 (r=.23) |
|  | Children  (Study 1/T1) | Independent groups | 23/15 | d = .84 (r=.39) |
|  | Children (Study 2) | Independent groups | 28/49 | d = .59 (r=.28) |
|  | Parents (overall) | Independent groups | 142/24 | d = .55 (r=.27) |
|  | Parents (Study 1/T1) | Independent groups | 59/8 | d = .95 (r=.43) |
|  | Parents (Study 2) | Independent groups | 82/17 | d = .68 (r=.32) |

Note: T1=baseline

Sensitivity analysis for Research Question 3: Detectable effect sizes for longitudinal change in Fear of Progression (FoP) from T1 to T2; dz = effect size for dependent/paired samples

| Sample | Statistical comparison | N | Sensitivity result |
| --- | --- | --- | --- |
| Children | Paired | 27 | dz = 0.56 |
| Parents | Paired | 41 | dz = 0.45 |

Sensitivity analysis for Research Question 4a: Parent–child differences in FoP within dyads;

dz = effect size for dependent/paired samples

| Study (Time point) | N (dyads) | Sensitivity result |
| --- | --- | --- |
| Study 1 (T1) | 37 | dz = 0.47 |
| Study 1 (T2) | 26 | dz = 0.57 |
| Study 2 | 73 | dz = 0.33 |

Note: T1=baseline, T2= one year follow-up

Sensitivity analysis for Research Question 4b: Parent–child correlations in FoP within dyads;

r = bivariate correlation coefficient

| Study (Time Point) | N (dyads) | Sensitivity result |
| --- | --- | --- |
| Study 1 (T1) | 37 | r = .44 |
| Study 1 (T2) | 26 | r = .52 |
| Study 2 | 73 | r = .32 |

Note: T1=baseline, T2= one year follow-up
